# Antinociceptive Action of Thymoquinone-Loaded Liposomes in an In Vivo Model of Tendinopathy

**DOI:** 10.3390/pharmaceutics15051516

**Published:** 2023-05-17

**Authors:** Laura Micheli, Lorenzo Di Cesare Mannelli, Elena Mosti, Carla Ghelardini, Anna Rita Bilia, Maria Camilla Bergonzi

**Affiliations:** 1 Department of Neuroscience, Psychology, Drug Research and Child Health—NEUROFARBA—Pharmacology and Toxicology Section, University of Florence, Viale G. Pieraccini 6, 50139 Florence, Italy; laura.micheli@unifi.it (L.M.); lorenzo.mannelli@unifi.it (L.D.C.M.); carla.ghelardini@unifi.it (C.G.); 2 Department of Chemistry Ugo Schiff, University of Florence, Via Ugo Schiff 6, Sesto Fiorentino, 50019 Florence, Italy; elena.mosti@stud.unifi.it (E.M.); ar.bilia@unifi.it (A.R.B.)

**Keywords:** thymoquinone, nociception, tendinopathy, liposomes, hyaluronic acid

## Abstract

Tendinopathies represent about 45% of musculoskeletal lesions and they are a big burden in clinics characterized by activity-related pain, focal tendon tenderness and intra-tendinous imaging changes. Many approaches have been proposed for tendinopathies’ management (e.g., nonsteroidal anti-inflammatory drugs, corticosteroids, eccentric exercises, laser therapy), unfortunately with very little support of efficacy or serious side effects, thus making the identification of new treatments fundamental. The aim of the study was to test the protective and pain reliever effect of thymoquinone (TQ)-loaded formulations in a rat model of tendinopathy induced by carrageenan intra-tendon injection (20 µL of carrageenan 0.8% on day 1). Conventional (LP-TQ) and hyaluronic acid (HA)-coated TQ liposomes (HA-LP-TQ) were characterized and subjected to in vitro release and stability studies at 4 °C. Then, TQ and liposomes were peri-tendon injected (20 µL) on days 1, 3, 5, 7 and 10 to evaluate their antinociceptive profile using mechanical noxious and non-noxious stimuli (paw pressure and von Frey tests), spontaneous pain (incapacitance test) and motor alterations (Rota rod test). Liposomes containing 2 mg/mL of TQ and covered with HA (HA-LP-TQ2) reduced the development of spontaneous nociception and hypersensitivity for a long-lasting effect more than the other formulations. The anti-hypersensitivity effect matched with the histopathological evaluation. In conclusion, the use of TQ encapsulated in HA-LP liposomes is suggested as a new treatment for tendinopathies.

## 1. Introduction

Tendon disorders are a class of pathologies that include traumatic injuries as well as chronic diseases, such as tendinopathy. Pain, swelling and functional limitations of the tendon and nearby anatomical structures are the main characteristics of tendinopathy [1]. Different pharmacological molecules and metabolic diseases can also be related to tendon pathologies. The pharmacological treatment of tendinopathies is difficult because tendons have a reduced vascularization with low drug availability in the target site. Furthermore, the difficulty lies in the extremely heterogeneous nature of tendinopathies and in their numerous symptoms [2]. Treatment options may include pharmacological and non-pharmacological approaches such as physiotherapy, exercise, anti-inflammatory drugs, corticosteroids, nitric oxide patches, growth factors and stem cells [3]. Despite the fact that non-steroidal anti-inflammatory drugs are the most commonly used, they present limitations due to gastrointestinal side effects, colitis and hepatic failure [3,4]. Furthermore, corticosteroids are effective in relieving pain, but they cause the individual to overexert a weakened tendon and they decrease the collagen synthesis [5,6]. Therefore, there is a need for new and alternative treatments that enhance healing and decrease the side effects. 

In the present work, we highlighted the beneficial potential of thymoquinone (TQ), a bioactive constituent of *Nigella sativa* L. [7] with antioxidant and anti-inflammatory activities. Other therapeutic properties are hepatoprotective, cardioprotective, anticancer, antidiabetic, anti-arthritic, neuroprotective and antimicrobial [8,9,10]. For these properties, TQ could represent a valid option for tendinopathies’ management. Nevertheless, it is hampered by pharmacokinetics characteristics such as short half-life, low biological stability, poor aqueous solubility and low bioavailability. Nanoformulations have gained remarkable attention to improve pharmacokinetics parameters and enhance the pharmacological activities of TQ [8,11,12,13]. TQ nanocarriers were effective in the treatment of arthritic inflammations: phospholipidic nanomatrix [14], topical ethosomes [15] and liposomal chitosan gel [16]. They enhanced the therapeutic efficacy of TQ as investigated in a carrageenan-induced paw inflammation model. 

In this study, liposomal formulations were proposed as a biocompatible drug delivery system to improve the solubility and bioactivity of TQ. 

For the first time, in this study, TQ conventional and hyaluronic acid (HA)-coated liposomes were evaluated in a model of tendinopathy. HA was considered for its physiological role in the homeostasis of tendons [17]. It is a natural polymer with anti-inflammatory effects on cells and tissues and positive effects on cell proliferation and collagen synthesis [18]. HA reduces the apoptosis of tendon-derived cells’ collagen type I protein secretion [19]. It has a beneficial effect on both the repair site and the synovial sheath, decreasing the peripheral inflammatory response and promoting contact healing [20]. The antioxidant properties of HA are reported [21,22].

From all these considerations, the antinociceptive action of conventional and HA-coated TQ liposomes was evaluated in the model of tendinopathy induced in rats by the intra-tendon injection of carrageenan. The formulations were physically and chemically characterized and subjected to in vitro release and stability studies.

## 2. Materials and Methods

### 2.1. Material

Egg phosphatidylcholine (Phospholipon 90G) was purchased from Lipoid AG, Cologne, Germany. Thymoquinone, cholesterol ≥ 95%, phosphate buffered saline (PBS 0.01 M) powder (29 mM NaCl, 2.5 mM KCl, 7.4 mM Na2HPO4·7H2O, 1.3 mM KH2PO4) pH 7.4 and Tween 80 were from Sigma Aldrich, Milan, Italy. Sodium hyaluronate (M.W. 1000 KDa, HA) was obtained from Altergon, Avellino, Italy. All the solvents used were HPLC grade from Sigma Aldrich, Milan, Italy. Water was purified by Millipore, Milford, MA, USA, Milli-Qplus system. Phosphotungstic acid (PTA) was from Electron Microscopy Sciences, Hatfield, PA, USA.

### 2.2. Preparation of Liposomal Formulations

TQ-loaded liposomes (LP-TQ) and HA-coated TQ liposomes (HA-LP-TQ) were prepared using the thin layer evaporation method [22]. TQ, egg phosphatidylcholine and cholesterol were dissolved in dichloromethane under sonication for 2 min. The solvent was evaporated, and the dry lipid film was hydrated with deionized water under mechanical stirring at 50 °C for 30 min. To reduce the size and improve the homogeneity of the sample, an ultrasound probe was applied for 2 min, with sonication intervals of 2 s and an intensity equal to 46%. The HA coating was achieved using the drop-wise method [23]. Two mL of 0.1% *w*/*v* solution of HA in deionized water was added to 2 mL of LP-TQ dispersion. The sample was subjected to magnetic stirring for 1 h at room temperature. Then, once the coating was completed, a sonication was performed using the ultrasonic probe for 1 min, with intervals of 0.5 s and an intensity of 46%. Two conventional liposomes containing 2 mg/mL (LP-TQ1) and 4 mg/mL (LP-TQ2) of TQ and two HA-coated liposomes containing 1 (HA-LP-TQ1) and 2 mg/mL (HA-LP-TQ2) of TQ were prepared. 

### 2.3. Characterization of Liposomes

Liposomes’ physical characterization was performed with light scattering (LS), using a Ζsizer Nano series ZS90 (Malvern Instruments, Malvern, UK) outfitted with a temperature controller set at 25 °C. The encapsulation efficiency (EE%) was determined by the dialysis bag method [24].

### 2.4. Storage Stability

The LP-TQ and LP-TQ-HA formulations were kept in the fridge at a temperature of +4 °C for 5 weeks. Each week, the dimensions of the samples (Size), the polydispersion index (PdI) and the Zeta potential (ZP) and encapsulation efficiency (EE%) were evaluated. 

### 2.5. In Vitro Release

The release study of TQ from the liposome in comparison to a saturated aqueous solution of TQ was carried out with the dialysis bag method using regenerated cellulose dialysis membranes (Spectrum Laboratories, Inc., Breda, The Netherlands, MWCO 3–4 kDa) [25]. Two mL of the liposomal formulation or solution was placed into dialysis membranes and immersed into 200 mL of Tween 80 (0.5% *w*/*v*) in PBS at 37 °C under magnetic stirring. At predetermined intervals, 1 mL of each release medium was withdrawn and replaced with an equal volume of the fresh solution. The TQ concentration in the samples was determined by an HPLC analysis [26]. All studies were performed in triplicate. 

The Korsmeyer–Peppas model, Hixson Crowell model, Higuchi model, first order and zero order mathematical models were applied to evaluate the mechanism of the TQ release from the formulations. The best fitted model was selected considering a high regression coefficient (R2) value for the release data.

### 2.6. Animals

For all the experiments described below, male Sprague-Dawley rats (Envigo, Varese, Italy) weighing approximately 200–250 g at the beginning of the experimental procedure were used. Animals were housed in CeSAL (Centro Stabulazione Animali da Laboratorio, University of Florence) and used at least one week after their arrival. Four rats were housed per cage (size 26 × 41 cm^2^), kept at 23 ± 1 °C with a 12 h light/dark cycle, light at 7 a.m. and were fed a standard laboratory diet and tap water ad libitum. All efforts were made to minimize animal suffering and to reduce the number of animals used.

### 2.7. Induction of Tendonitis and Treatments

The animals were anesthetized with isoflurane (4% for induction and 1.5% for maintenance of anesthesia). Tendon damage was induced near the osteotendinous junction of the rat’s right Achilles tendon by a single percutaneous injection of 20 μL of carrageenan 0.8%, after having flexed the paw to form a 45° angle, using a 30 G needle on day 1. Carrageenan was solubilized in a physiological solution. The control animals were treated with saline solution [27,28]. Twenty μL of HA-LP-TQ1 (1 mg/mL) or HA-LP-TQ2 (2 mg/mL), HA-LP, LP-TQ1 (2 mg/mL) formulations and a saturated aqueous solution of TQ (0.55 mg/mL) were peri-tendon injected on days 1, 3, 5, 7 and 10. Behavioral measurements were performed on days 3, 5, 7, 10 and 13 before the new daily treatment with the formulations.

### 2.8. Paw Pressure Test

The nociceptive threshold in the rat was determined with an analgesimeter (Ugo Basile, Varese, Italy). Briefly, a constantly increasing pressure was applied to a small area of the dorsal surface of the hind paw using a blunt conical mechanical probe. Mechanical pressure was increased until vocalization or a withdrawal reflex occurred while rats were lightly restrained. Vocalization or withdrawal reflex thresholds were expressed in grams. These limits assured a more precise determination of the mechanical withdrawal threshold in experiments aimed to ascertain the effect of treatments. An arbitrary cut-off value of 100 g was adopted. The data were collected by an observer who was blinded to the protocol [29,30].

### 2.9. Von Frey Test

Mechanical allodynia was measured using an electronic von Frey apparatus as previously reported [31,32]. 

### 2.10. Incapacitance Test

Weight-bearing changes were measured using an incapacitance apparatus (Linton Instrumentation, Norfolk, UK) to detect changes in the postural equilibrium after a hind limb injury [33,34]. The method used was previously described by Di Cesare Mannelli and colleagues [35]. Data were expressed as the difference between the weight applied to the limb contralateral to the injury and the weight applied to the ipsilateral one (Δ weight).

### 2.11. Beam Balance Test

A balance beam test [36] consisted of the rats being placed on a narrow strip of wood (30 cm × 1.3 cm) while balancing, and the scoring standards were as follows: 0 point, the four limbs were all on the wood in a balance situation; 1 point, limbs of one side were able to grasp the wood or shake on the wood; 2 points, one or two limbs slipped from the wood; 3 points, three limbs slipped from the wood; 4 points, suspended on the wood and fell over after struggle [37].

### 2.12. Rota Rod Test

The Rota rod apparatus (Ugo Basile, Varese, Italy) consisted of a base platform and a rotating rod with a diameter of 6 cm and a non-slippery surface and was used as a measure of motor coordination. Full details were reported in a previous paper by Micheli and colleagues [28].

### 2.13. Histological Evaluation

At the end of the behavioral evaluations, animals were sacrificed, and tendons collected for the histological analysis. Briefly, tendon samples were fixed in buffered 4% formalin, dehydrated and embedded in paraffin (Bio-Optica, Milan, Italy). Ten µm thickness longitudinal sections were processed for Hematoxylin-Eosin (Bio-Optica, Milan, Italy), as previously described [28]. 

Five slides for each tendon were randomly selected and examined by two blinded investigators under an optical microscope. The slides were interpreted using a modified histological grading score from Kihara and colleagues [38], composed of various parameters: extracellular matrix organization (0–2), tissue homogeneity (0–2), presence of degenerative changes (0–2), cell nucleus morphology (0–2), cell distribution (0–2) and alignment (0–2), vascularization (0–1), inflammation (0–1) and Azan-Mallory red stain intensity (0–2). The total score for each animal ranged between 0 (most severe tendon impairment) and 16 points (control, normal tendon). Demonstrative images were acquired at 10× and 40× total magnification by a Nikon light microscope (Nikon Olympus BX40, Tokyo, Japan).

### 2.14. Statistical Analysis

All the pharmacological experimental procedures were performed by researchers blinded to the treatments. Each value represented the mean ± S.E.M of eight rats per group, performed in two different experimental sets. The analysis of variance was performed by an analysis of variance (ANOVA). Bonferroni’s significant difference procedure was used as a post hoc comparison. p values of less than 0.05 were considered significant. Data were analyzed using the ‘Origin 9’ software (OriginLab, Northampton, MA, USA).

## 3. Results and Discussion

### 3.1. Preparation and Characterization of Liposomes

Liposomes were selected as the drug delivery system to improve TQ solubility and realize a biocompatible formulation to test in the in vivo model of tendinopathy. The optimized formulation (LP-TQ) contained phosphatidylcholine and cholesterol in a 4:1 weight ratio, and it was able to load 2 and 4 mg/mL of TQ, improving until the eight-fold solubility. The optimized systems had good physical and chemical parameters (Table 1). Then, the formulations were coated with HA (0.1% *w*/*v*), obtaining two preparations with a final TQ concentration of 1 mg/mL (LP-TQ-HA1) and 2 mg/mL (LP-TQ-HA2). Both coated liposomes maintained good values of sizes, PdI, ZP and EE% (Table 1). The increase in the particle sizes and the change of ZP confirmed the HA deposition [39].

### 3.2. Stability Studies

**Figure 1:**
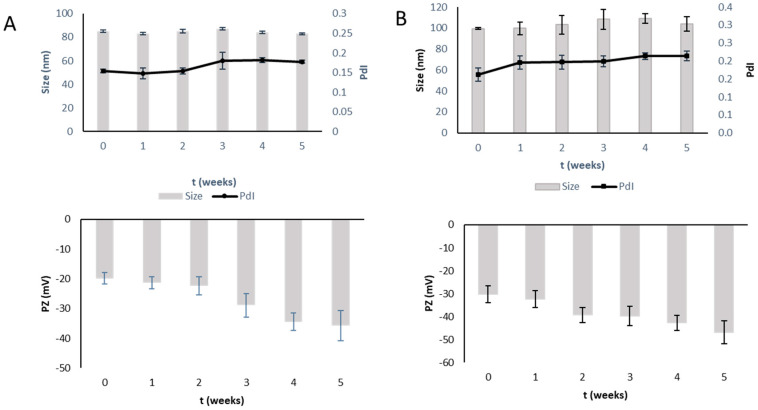
Storage stability test of TQ-loaded liposomes at +4 °C for 5 weeks. (**A**) LP-TQ1; (**B**) LP-TQ2 (mean ± SD, *n* = 3).

**Figure 2:**
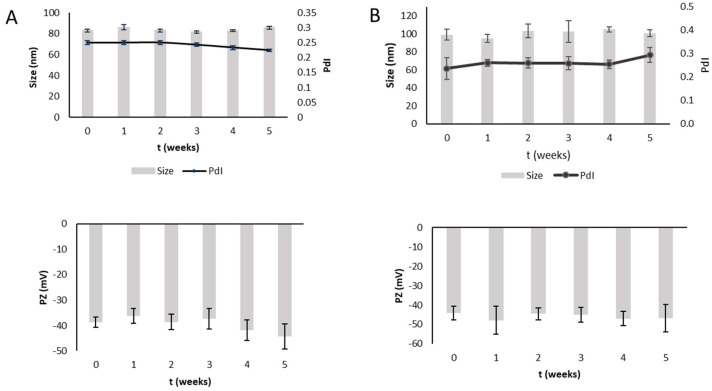
Storage stability test of HA-coated liposomes at +4 °C for 5 weeks. (**A**) HA-LP-TQ1; (**B**) HA-LP-TQ2 (mean ± SD, *n* = 3).

The liposomal formulations were kept for 5 weeks at +4 °C. The physicochemical properties were checked by monitoring particle sizes, PdI, ZP and drug entrapment. All dispersions showed a high level of physical stability (Figure 1 and Figure 2), as evidenced by small changes in sizes and PdI values. A little increase in ZP was observed. EE% ranged from 73 ± 3 to 71 ± 3 for LP-TQ1 and from 67 ± 4 to 54 ± 1 for LP-TQ2, probably due to the higher amount of TQ in the formulation that could destabilize the formulation after 5 weeks. Both liposomes showed good stability as dispersions for at least 4 weeks.

The HA coating kept the stability of the systems, improving the physical one, as evidenced by the constant values of ZP. The EE% for LP-TQ-HA1 was from 70 ± 2 to 66 ± 2 and in the case of LP-TQ-HA2 was from 65 ± 5 to 62 ± 3.

### 3.3. In Vitro Release Study

**Figure 3:**
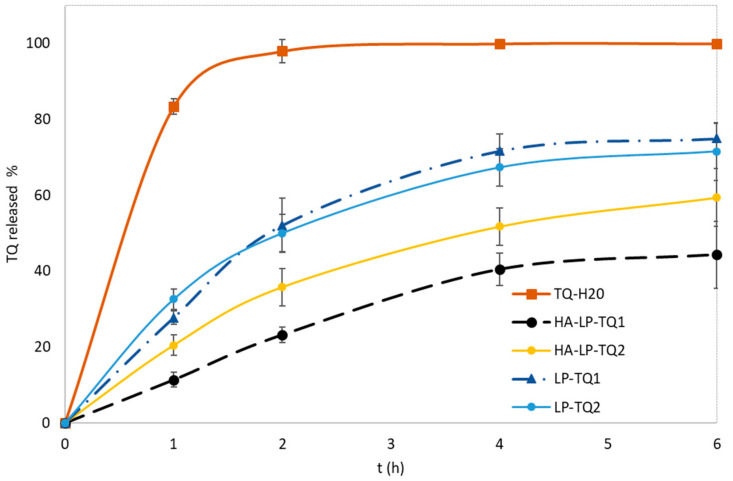
In vitro release profiles of TQ from the uncoated and HA-coated liposomes and TQ aqueous saturated solution in phosphate buffered saline (PBS) medium containing Tween 80 (0.5% *w*/*v*) at pH 7.4, at 37 °C. (Mean ± SD, *n* = 3).

The release profile of TQ from liposomes was compared with the release of a saturated aqueous solution at 37 °C (Figure 3). Both the HA-coated and uncoated formulations did not prevent the release of TQ. In the aqueous solution, TQ was completely released in about two hours, while the percentage was 71% and 74% from LP-TQ1 and LP-TQ2, respectively, and 44% and 59% from HA-LP-TQ1 and HA-LP-TQ2, respectively. Both liposomal formulations realized a prolonged release with respect to the aqueous solution, and a slower release than the uncoated liposomes was obtained in the case of the HA coating.

The release data were fitted to the appropriate mathematical models (zero order, first order, Higuchi, Korsmeyer–Peppas and Hixson–Crowell; Table 2) for the LP-TQ1 and HA-LP-TQ2 liposomes tested in the in vivo model of tendinopathy.

Comparing the values of the regression coefficient of the release curves of LP-TQ1 and HA-LP-TQ2 reported in Table 2, the Higuchi model (K_H_ = 27.07, R^2^ = 0.952 and K_H_ = 19.18, R^2^ = 0.977, respectively; Table 2) was the best one to describe the kinetics of these two liposomes, as also reported in the literature [24,40]. Higuchi described the drug release as a diffusion process based on Fick’s law, and a linear relationship was found between the amount of drug released and the square root of time (Q = K_H_t^1/5^). It could be concluded that the vesicles acted as reservoir systems for the continuous delivery of the encapsulated drug. This suggests that the TQ release is mainly driven by a diffusion-controlled mechanism [41].

### 3.4. In Vivo Study

The TQ formulations were developed with a view of finding new and alternative anti-inflammatory treatments of tendinopathy. It has been previously reported that TQ is involved in the regulation of various molecular signaling pathways. The molecule also reduces ROS production by inhibiting the expression of various pro-inflammatory factors, including IL-1β, IL-6, TNF-α, IFN-γ and PGE2 [42]. For these properties, TQ could represent a valid option for tendinopathies’ management. Nevertheless, its pharmacological relevance is hampered by its poor aqueous solubility and low bioavailability. The efficacy of the liposomal formulations was then tested in a rat model of tendinopathy. The tendinopathy was induced by a single intra-tendon injection of 20 μL of carrageenan 0.8% on day 1. The efficacy of different liposomal formulations containing TQ was evaluated following five peri-tendon treatments performed on days 1, 3, 5, 7 and 10. Behavioral measurements were performed in the time course on days 3, 5, 7, 10 and 13 to highlight the pain threshold of the animals in response to noxious and non-noxious stimuli (paw pressure and von Frey tests, respectively), to evaluate the spontaneous nociception (Incapacitance test) and the motor skills (beam balance test) and coordination (Rota rod test). Peri-tendinous administration is usually used for the management of chronic tendinopathies [43]. This modality of administration avoids injecting the drug directly into the tendon, a procedure that might lead to tendon damage and rapture. The very low bioavailability of TQ has reduced the studies that explore the clinical application of this compound due to its low oral absorption. To overcome this problem, nanotechnology is usually pursued to improve TQ bioavailability. For this purpose, we tested TQ-loaded liposomes (LP-TQ) and HA-coated TQ liposomes (HA-LP-TQ) in comparison to TQ alone and the nanocarrier HA-LP alone. It has been already demonstrated that the topical and oral administration of *Nigella sativa* oil had antinociceptive properties against different stimuli in mice and that these effects are related to TQ. The TQ antinociceptive effect could be due to a direct interaction with opioid receptors; specifically, μ and κ opioids’ receptors subtypes at the supraspinal levels are involved [44]. From a review of the literature, it has also emerged how *Nigella sativa* and TQ can be promising therapeutic approaches for the management of articular pain with positive feedback from pre-clinical [45,46] as well as clinical studies [47,48] with mostly recognized anti-inflammatory and anti-oxidant effects. 

**Figure 4:**
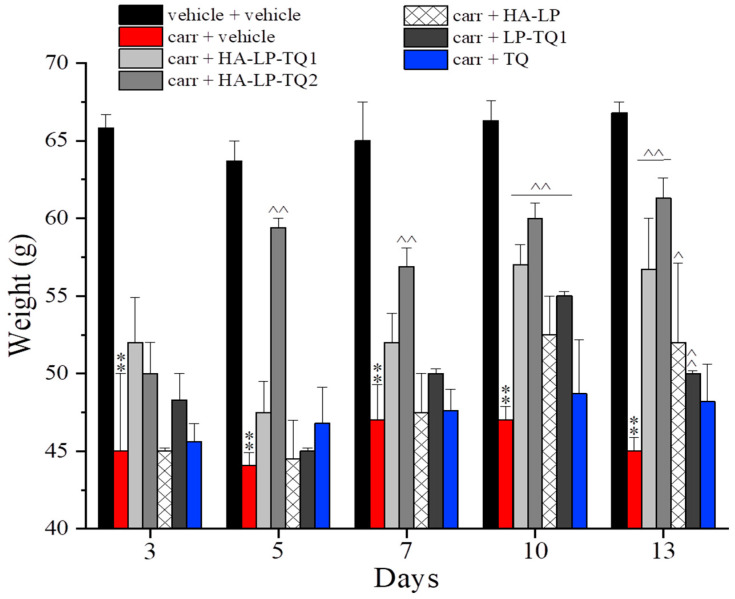
Antinociceptive properties of liposomes against carrageenan-induced mechanical hyperalgesia. Following intra-tendon injection of 20 µL of carrageenan 0.8% on day 1, the efficacy of peri-tendon injections (20 µL) of HA-LP-TQ1 (1 mg/mL), HA-LP-TQ2 (2 mg/mL), HA-LP, LP-TQ1 (2 mg/mL) and TQ (0.55 mg/mL) was evaluated. Formulations were injected on days 1, 3, 5, 7 and 10 and the response to a noxious mechanical stimulus was assessed by the paw pressure test in time course (days 1, 3, 5, 7, 10 and 13). Control animals were treated with vehicles. The value represents the mean of 8 rats performed in two different experimental sets. ** *p* < 0.01 compared to the vehicle + vehicle group; ^ *p* < 0.05 and ^^ *p* < 0.01 compared to the carrageenan + vehicle group.

The injection of carrageenan significantly lowered the animal’s nociception threshold in response to a noxious stimulus (paw pressure test) starting on day 3 from the damage and up to day 13 (Figure 4). The peri-tendon injections of HA-LP-TQ2 were significantly effective, increasing the weight burdened by the animal on the ipsilateral paw starting from day 5 (after two treatments) and remaining effective up to the end of the experiment (Figure 4). The lower dose of 1 mg/mL contained in the HA-LP-TQ1 was effective after four treatments (day 10). A reduction of mechanical hypersensitivity was also recorded on days 10 and 13 with HA-LP and LP-TQ1, albeit with less effectiveness in comparison to HA-LP-TQ1 and HA-LP-TQ2. The treatment with only TQ was ineffective, thus confirming the necessity to deliver the TQ in the liposomal formulations to evoke an antinociceptive effect. The HA was considered as a constituent of the liposomal formulation, since it is one of the fundamental components of tendon tissue and it is able to enhance the cellular activities of fibroblasts, including their adhesivity, extracellular matrix synthesis and proliferation [49]. Among the therapeutic strategies such as platelet-rich plasma (PRP), adipose-derived mesenchymal stromal cells and botulinum toxin, HA injections seem to inhibit the pro-inflammatory response by local fibroblast [50], improve function, reduce pain and reduce tendon rubbing in pre-insertion areas during major tendinopathies and post-surgical tendon repair [51]. Its positive effect as a pain reliever was confirmed in the HA-LP-treated animals in which an anti-hyperalgesic effect was highlighted on day 13 even in the absence of TQ.

**Figure 5:**
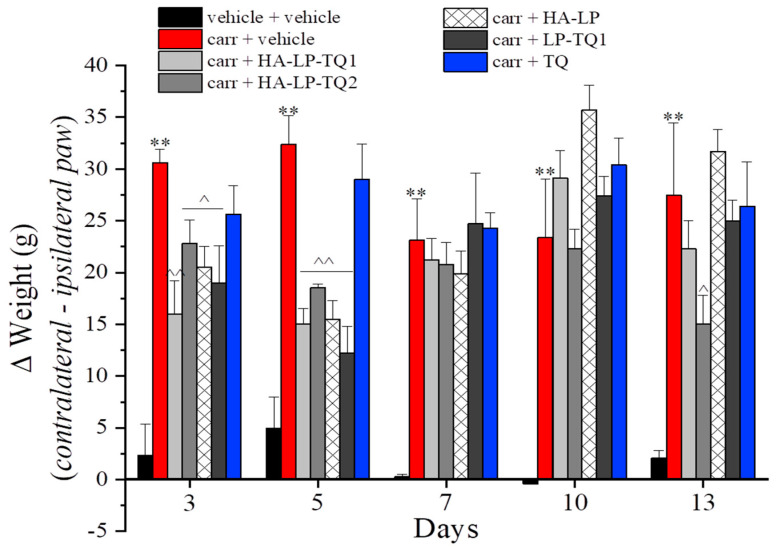
Antinociceptive properties of liposomes against carrageenan-induced spontaneous nociception. Following intra-tendon injection of 20 µL of carrageenan 0.8% on day 1, the efficacy of peri-tendon injections (20 µL) of HA-LP-TQ1 (1 mg/mL), HA-LP-TQ2 (2 mg/mL), HA-LP, LP-TQ1 (2 mg/mL) and TQ (0.55 mg/mL) was evaluated. Formulations were injected on days 1, 3, 5, 7 and 10 and the development of spontaneous nociception was assessed by the Incapacitance test in time course (days 1, 3, 5, 7, 10 and 13). Control animals were treated with vehicles. The value represents the mean of 8 rats performed in two different experimental sets. ** *p* < 0.01 compared to the vehicle + vehicle group; ^ *p* < 0.05 and ^^ *p* < 0.01 compared to the carrageenan + vehicle group.

The carrageenan injection was also able to generate the hind limb weight-bearing alteration measured as spontaneous nociception by the Incapacitance test (Figure 5). Postural unbalance was higher in carrageenan-treated animals in comparison to the control group (vehicle + vehicle) in all evaluations performed. All treatments reduced the difference between the weight placed by the animal on the contralateral and the ipsilateral paw (expressed as Δ weight) on day 3 (after one injection of each formulation); on this day, the HA-LP-TQ1 treatment showed the highest efficacy. The same trend was confirmed by the behavioral evaluations performed on day 5. All treatments lost their efficacy over the next days with the exception of HA-LP-TQ1, which was still active on day 13. As already shown in the paw pressure test, the only aqueous solution of TQ was ineffective in reducing spontaneous pain (Figure 5).

**Figure 6:**
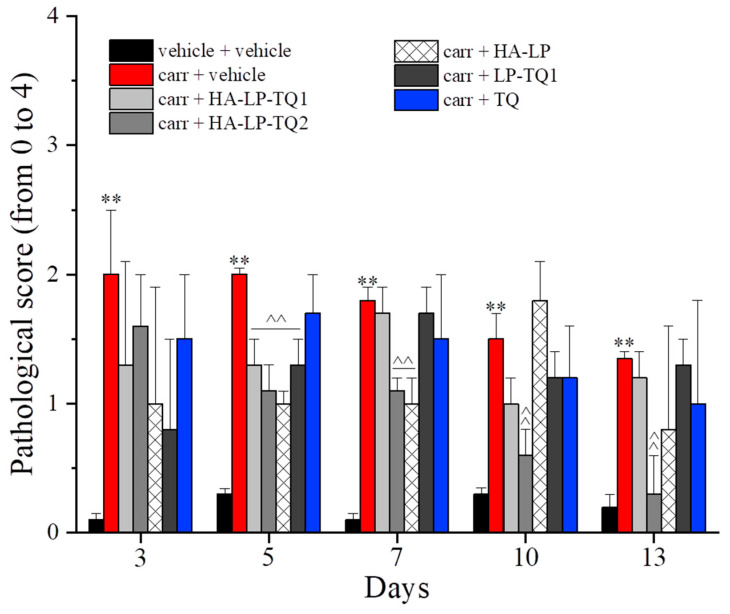
Antinociceptive properties of liposomes against carrageenan-induced motor alterations. Following intra-tendon injection of 20 µL of carrageenan 0.8% on day 1, the efficacy of peri-tendon injections (20 µL) of HA-LP-TQ1 (1 mg/mL), HA-LP-TQ2 (2 mg/mL), HA-LP, LP-TQ1 (2 mg/mL) and TQ (0.55 mg/mL) was evaluated. Formulations were injected on days 1, 3, 5, 7 and 10 and the efficacy against motor alterations was assessed by the beam balance test in time course (days 1, 3, 5, 7, 10 and 13). Control animals were treated with vehicles. The value represents the mean of 8 rats performed in two different experimental sets. ** *p* < 0.01 compared to the vehicle + vehicle group; ^^ *p* < 0.01 compared to the carrageenan + vehicle group.

**Figure 7:**
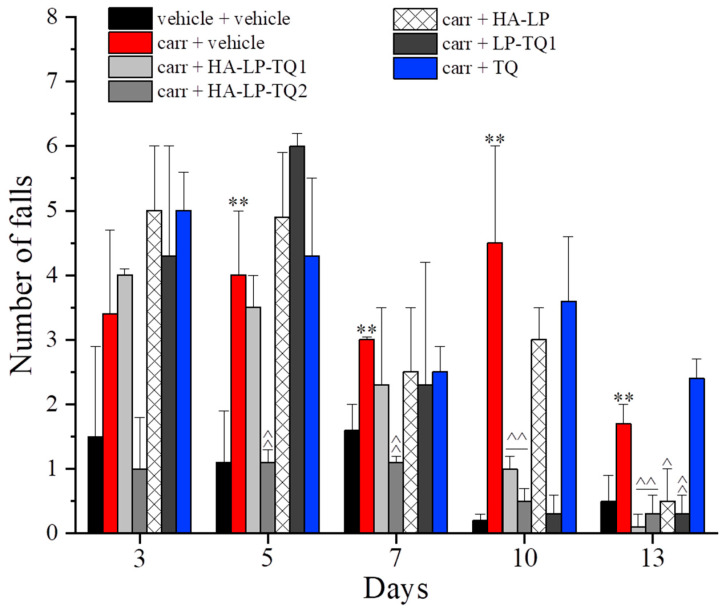
Antinociceptive properties of liposomes against carrageenan-induced motor alterations. Following intra-tendon injection of 20 µL of carrageenan 0.8% on day 1, the efficacy of peri-tendon injections (20 µL) of HA-LP-TQ1 (1 mg/mL), HA-LP-TQ2 (2 mg/mL), HA-LP, LP-TQ1 (2 mg/mL) and TQ (0.55 mg/mL) was evaluated. Formulations were injected on days 1, 3, 5, 7 and 10 and the efficacy against motor alterations was assessed by the Rota rod test in time course (days 1, 3, 5, 7, 10 and 13). Control animals were treated with vehicles. The value represents the mean of 8 rats performed in two different experimental sets. ** *p* < 0.01 compared to the vehicle + vehicle group; ^ *p* < 0.05 and ^^ *p* < 0.01 compared to the carrageenan + vehicle group.

Carrageenan also affected the motor skills and coordination of the rats as depicted in Figure 6 and Figure 7. The carrageenan-treated animals showed an increase in the pathological score, in comparison to the control group, that lasted up to day 13. HA-LP-TQ2 significantly improved the animals’ motor skills, halving the score assigned from day 5 to day 13 and showing the best treatment in relieving motor alterations evoked by carrageenan (Figure 6). HA-LP-TQ2 was also effective in reducing the number of falls from the rotating rod that were upregulated in carrageenan + vehicle-treated animals. On day 10, HA-LP-TQ1 and LP-TQ1 were effective, and on day 13, all these treatments were able to reduce this parameter. On the contrary, TQ alone proved ineffective at all time points (Figure 7). 

**Figure 8:**
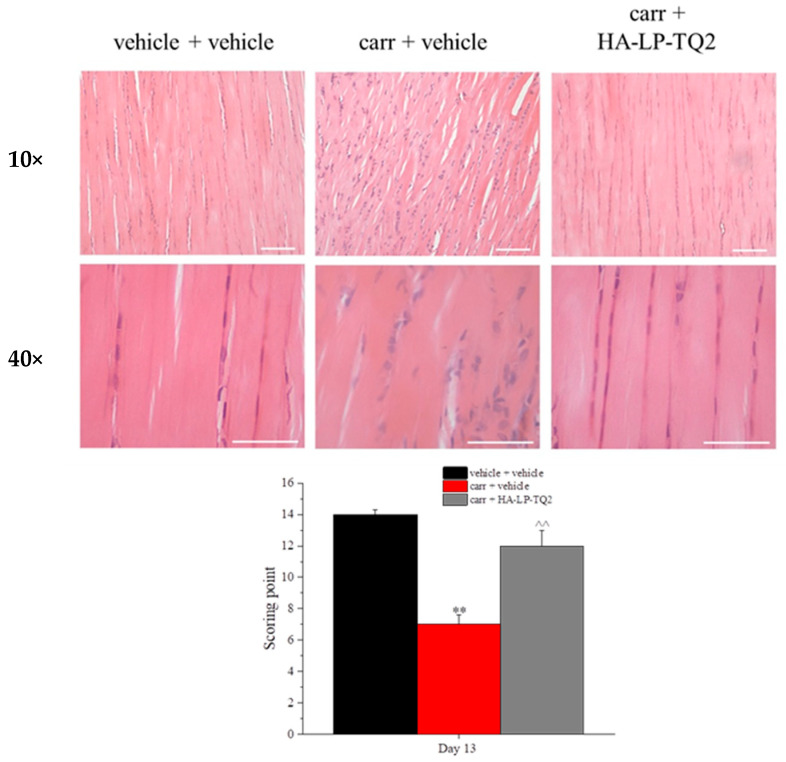
Histological evaluation of HA-LP-TQ2 injections on tendinopathy models. After 5 peri-tendon treatments with HA-LP-TQ2 on carrageenan, damaged tendon samples of the animals were collected. To make histological evaluation, Hematoxylin-Eosin was performed. The histological score was calculated according to the following parameters: extracellular matrix organization (0–2), tissue homogeneity (0–2), presence of degenerative changes (0–2), cell nucleus morphology (0–2), cell distribution (0–2) and alignment (0–2), vascularization (0–1), inflammation (0–1) and Azan-Mallory red stain intensity (0–2). Total score for each animal ranged between 0 (most severe tendon impairment) and 16 points (control, normal tendon). ** *p* < 0.01 compared to the vehicle + vehicle group; ^^ *p* < 0.01 compared to the carrageenan + vehicle group.

At the end of the behavioral evaluations, a histological analysis of the tendons was performed. Based on the in vivo results, the analysis was conducted on the most promising treatment, which turned out to be HA-LP-TQ2. The tendons of the control animals appeared normal with well-aligned parallel and compact collagen fibers as shown in Figure 8. The damage with carrageenan determined a disorganization of the tendon matrix represented by discontinuous, crimped and thinned collagen fibers in comparison to the control group. Moreover, an increase in vascularity was also evident by a moderate increase in the smaller capillaries. HA-LP-TQ2 peri-tendon treatments partially restored the degenerative changes caused by carrageenan (Figure 8). The healing process of tendons can be summarized with three different phases that overlap with each other: inflammation, formative and remodeling phases. The high production of collagen type I is mandatory for rapid and effective tendon healing [52]. It has been demonstrated that TQ can regulate the expression of the mitogen-activated protein kinase (MAPKs), p-p38, p-JNK, p-ERK and PI3K/pAkt, thus upregulating collagen I expression in a dose-dependent manner [53,54]. That would explain the protective and regenerative effect of TQ previously highlighted by Soltanfar and colleagues on an experimental model of tendinopathy in rabbits induced by a traumatic injury, which demonstrated a pronounced effect exerted by TQ treatment on collagen production [55]. The results that we obtained in our study were also in line with previous evidence in which the protective effect of *Nigella sativa* extract containing TQ and other bioactive compounds was highlighted in a rat model of Achilles tendon rupture. Ruru and colleagues indeed demonstrated a stronger tensile strength of tendons and lower malondialdehyde levels in rats treated with the extract, suggesting this treatment as adjuvant for tendon rupture therapy [56]. The promising results achieved in our study could be due also to the anti-inflammatory properties of TQ already known and reported by the scientific literature [57,58] and not only to a direct action of collagen production. In particular, TQ can inhibit pro-inflammatory cytokines that are involved in collagen synthesis and MMPs activation that lead to collagen degradation [59,60]. Not to be excluded, a mechanism of action of TQ relates to an antioxidant activity which can protect fibroblasts from reactive oxygen species overproduced in a damaged tendon tissue.

In the literature, several TQ liposome formulations have been successfully developed to work against colon, prostate, cervical, skin and breast cancer [61,62,63,64,65], resulting in an enhanced solubility and improved therapeutic efficacy of TQ. TQ also showed a synergistic cytotoxicity and ameliorated the encapsulation efficiency when co-loaded into liposomes with docetaxel or curcumin [63,66]. In addition, TQ liposomes exhibited antibacterial and antifungal properties [67,68,69]. There are no examples in the literature of TQ HA-coated liposomes, except for two studies by the authors for the ocular delivery of TQ. Liposomal formulations, and, in particular, HA-coated liposomes, reduced the toxicity of TQ at high doses in HCE-2 and HConEC cells and improved the absorption at the nucleus level, with a more pronounced effect for HA-coated liposomes [26,39]. Furthermore, liposomes were effective in dry eye disease. Here, for the first time, the authors evaluated liposomal formulations in an in vivo model of tendinopathy. Liposomes improved the TQ solubility until eight folds and showed an antinociceptive activity in the in vivo test, with respect to free TQ, which was ineffective, as evidenced by the promising results. Furthermore, the HA coating was considered due to its physiological role in tendons [17]. HA improved the anti-hypersensitivity effect of TQ with respect to the uncoated formulations and prolonged the effect up to the end of the treatment. Liposomes containing 2 mg/mL of TQ and covered with HA (HA-LP-TQ2) reduced the development of spontaneous nociception and hypersensitivity for a long-lasting effect more than the other formulations. Furthermore, HA-LP-TQ2 partially restored the degenerative modifications caused by inflammation as evidenced by the histological findings.

## 4. Conclusions

The protective and pain reliever effects of TQ-loaded formulations in a rat model of tendinopathy induced by carrageenan intra-tendon injection have been demonstrated. Both conventional and HA-coated TQ liposomes improved the TQ solubility, guaranteed a prolonged release and were stable for 1 month at +4 °C. The HA coating enhanced and extended the anti-inflammatory effect of TQ, compared to the uncoated formulation and TQ alone. In particular, the liposomes containing the highest dose (2 mg/mL) of TQ and covered with HA (HA-LP-TQ2) reduced the development of spontaneous nociception and hypersensitivity for a long-lasting effect more than the other formulations. The anti-hypersensitivity effect matched with the histopathological evaluation, since this formulation partially restored the tendon damage evoked by carrageenan, thus improving the matrix organization and normalizing collagen fibers’ orientation. The promising results achieved in the study confirmed the protective and regenerative effects of TQ in addition to its anti-inflammatory properties and its action on collagen production. The liposomes represented a biocompatible formulation indispensable for the delivery of effective doses of TQ through injection. The HA coating too had a positive effect as a pain reliever. In conclusion, the use of TQ encapsulated in HA-LP is suggested as a new injection treatment for tendinopathy management.

## Figures and Tables

**Table 1:** Physical and chemical characterization of conventional (LP-TQ) and HA-coated liposomes of TQ, (mean ± SD, *n* = 3). PdI: polydispersion index; PZ: Zeta potential; EE%: encapsulation efficiency.

Sample	TQ mg/mL	Size (nm)	PdI	PZ (mV)	EE%
LP-TQ1	2	82 ± 1	0.15 ± 0.01	−20 ± 1	73 ± 3
LP-TQ2	4	96 ± 1	0.16 ± 0.01	−30 ± 2	67 ± 4
HA-LP-TQ1	1	88 ± 2	0.25 ± 0.01	−38 ± 1	70 ± 2
HA-LP-TQ2	2	99 ± 5	0.23 ± 0.01	−44 ± 2	65 ± 5

**Table 2:** Regression coefficient (R2) obtained in different kinetics models for TQ release from LP-TQ and HA-LP-TQ2.

Release Kinetics	LP-TQ1	HA-LP-TQ2
Zero order	0.756	0.930
First order	0.854	0.935
Korsmeyer–Peppas	0.423	0.686
Hixson–Crowell	0.820	0.944
Higuchi	0.952	0.977

## Data Availability

The data presented in this study are available on request from the corresponding author.
